# The miR-34 family and its clinical significance in ovarian cancer

**DOI:** 10.7150/jca.33831

**Published:** 2020-01-13

**Authors:** Hannah Welponer, Irina Tsibulak, Verena Wieser, Christine Degasper, Giridhar Shivalingaiah, Sören Wenzel, Susanne Sprung, Christian Marth, Hubert Hackl, Heidelinde Fiegl, Alain G. Zeimet

**Affiliations:** 1 Department of Obstetrics and Gynecology, Medical University of Innsbruck, Innsbruck, Tyrol, 6020, Austria; 2 Division of Human Genetics, Medical University of Innsbruck, Innsbruck, Tyrol, 6020, Austria.; 3 Institute of Pathology, Medical University of Innsbruck, Innsbruck, Tyrol, 6020, Austria.; 4 Biocenter, Division of Bioinformatics, Medical University of Innsbruck, Innsbruck, Tyrol, 6020, Austria.

**Keywords:** ovarian cancer, miR-34, p53, survival, carcinogenesis

## Abstract

The tumor suppressor miR-34 family is transcriptionally induced by p53. Clinical significance of the various miR-34 family members has not been studied in ovarian cancer. In 228 ovarian cancers and in 19 non-neoplastic fallopian tube samples we analysed miR-34 a/b/c expression in relation to clinicopathological characteristics and clinical outcome. We found significantly lower levels of miR-34 a/b/c in ovarian cancers as compared to control-tissues (*P*=0.002, *P*<0.001, *P*<0.001, respectively). Expression of miR-34 b/c revealed an inverse correlation with *BRCA1/2* mRNA-expression (BRCA1: miR34 b/c *P*=0.002 each; BRCA2: miR-34 b/c P<0.001 each), the same was true for miR-34a and *BRCA2* mRNA-expression (*P*<0.001). The miR-34 family expression was found to be significantly lower in type 2 in comparison to type 1 cancers (P<0.001) and in *TP53*-mutated compared with *TP53*-wild-type ovarian cancers (*P*<0.001, *P*=0.002, *P*=0.004, respectively). When low grade serous ovarian cancers were compared with high grade serous cancers the respective miR-34 a/b/c expression was 2.6-, 40.8- and 32.3-fold higher. The expression of each of the miR-34 family members was revealed to be of independent prognostic relevance regarding progression free survival (PFS); miR-34a: HR 0.6, *P*=0.033; miR-34b: HR 0.2, *P*=0.001 and miR-34c: HR 0.3, *P*=0.002, respectively). For overall survival (OS) independency of the prognostic value was confined to miR-34b (HR 0.4, *P*=0.016) and miR-34c (HR 0.6, *P*=0.049). The independency of the prognostic value of our identified thresholds was confirmed for PFS for miR-34c in a publicly available dataset (NCBI Gene Expression Omnibus GSE73582). Our findings suggest that downregulation of miR-34 family is a crucial part in ovarian cancer development. Low miR-34 levels are linked to a worse overall survival and progression free survival and may indicate a more aggressive disease.

## Introduction

Mortality due to ovarian cancer is considered to be one of the highest among malignancies in females 1. In advanced ovarian cancer surgery, with the aim of a complete clearance from all macroscopic tumor, and subsequent chemotherapy are the essential parts of treatment 2. Despite appropriate treatment, ovarian cancer will recur in more than 75% of the patients 3, 4.

Ovarian cancer can be classified into two major subgroups founded on two distinct backgrounds in carcinogenesis: Type 1 carcinomas develop from precursor lesions such as atypical proliferative tumors, generally grow slowly and tend to be restricted to the ovary at time of diagnosis 5. Frequently these cancers are associated with mutations of *BRAF* and *KRAS* 6. However, the largest group of cancers are the more aggressive type 2 tumors which originate from the fimbrial epithelium of the fallopian tube and develop through STICs (serous tubal intraepithelial carcinomas) as precursor lesions, from where malignant cells exfoliate into the free abdominal cavity and implant on the ovary and on the whole peritoneal surface. Mutations in *TP53* are an early event in the genesis and represent the leading driver of these high grade cancers 5, 7, 8.

The transcription factor p53 also known as the “guardian of the genome”, acts as a tumor suppressor 9-11. New insights into the way of function of p53 emerged that some mutations are able to confer even pro-oncogenic properties to the altered p53 protein 12, 13.

Furthermore, there is evidence that p53 transactivates microRNAs (miRNAs) of the 34 family. These miRNAs have been found to interfere with the mRNA of crucial cellular proliferative and anti-apoptotic regulators and negatively control their expression and thus support cell-cycle arrest, senescence and apoptosis 11, 14-18. Of special interest is that within the miR-34 family, miR-34a is encoded by a different gene than miR-34b and 34c, which both are encoded by a common gene 11.

These tumor-suppressing properties of this p53-miR34 interplay are of special importance during p53-detected DNA damage. Mutation of *TP53* consecutively may favor carcinogenesis and tumor proliferation by reduced levels of intracellular miR-34 family members 11, 17. Besides its p53 regulation, miR-34a and mir-34 b/c have also been found to be epigenetically regulated via CpG methylation of their promoter 19.

As *TP53* mutation represents the driver mutation in more than 95% of the high grade serous cancers, but is very uncommon in type 1 ovarian cancers 5, 7, we wanted to explore how far the members of the miR-34 family are involved in the carcinogenesis and biology of ovarian cancer. Therefore, differences between miR-34 expression profile in type 1 and type 2 cancers as well as in *TP53*-mutated and -wild-type ovarian cancers were of particular interest. Here we additionally performed also the first comprehensive survival analysis of the miR34-family in ovarian cancer.

## Material and Methods

### Study population

Ovarian tissue samples from 228 patients with ovarian cancer (OC) obtained at primary debulking (patients were 24 to 90 years old; median age at diagnosis was 61 years) and non-neoplastic tubal tissues from 19 patients obtained by elective salpingo-oophorectomy for benign conditions (patients were 30 to 73 years old, median age: 50 years) were collected and processed at the Department of Obstetrics and Gynecology of the Medical University of Innsbruck between 1989 and 2014 as described recently 20. We included all ovarian cancer patients where fresh frozen tissues were collected and sufficient material for RNA extraction was available. Systemic treatment of OC patients consisted of six adjuvant cycles of platinum-based chemotherapy. Written informed consent was obtained from all patients before enrolment. The study was reviewed and approved by the Ethics committee of the Medical University of Innsbruck (reference number: AN2015-0038 346/4.17) and conducted in accordance with the Declaration of Helsinki. The median observation period of all patients was 30 months (1 to 252 months) regarding the progression free survival and 59 months (1 to 289 months) concerning the median overall survival. Clinicopathological characteristics are shown in Table 1.

### Validation cohort

Gene expression data from two independent cohorts of OC patients were used for the validation of our findings (NCBI Gene Expression Omnibus; accession number GSE73583 (GSE73581 (OC179) and GSE73582 (OC133); 21). Patients without surgical treatment or patients with borderline tumors were excluded.

### RNA isolation, Reverse transcription and real-time PCR analysis

Total cellular RNA extraction was done as previously described 20. Reverse transcription was performed using the TaqMan™ MicroRNA Reverse Transcription Kit according the manufacturer's instructions (Applied Biosystems, Carlsbad, USA, Cat.no. 4366597).

TaqMan microRNA assays specific for miR-34a-5p, miR-34b-5p and miR-34c-5p respectively (Applied Biosystems, Assay ID 000426; ID 00427, and ID 00428 respectively) were used. miR-34 expression was normalized to RNU6B (Applied Biosystems, Assay ID 001093) using the standard curve method. The assays were performed in accordance with manufacturer's instructions using the QuantStudio 6 Flex system (Applied Biosystems).

### Mutation analysis

Genomic DNA from pulverized, quick-frozen OC specimens was isolated using the DNeasy tissue-kit (Qiagen, Hilden, Germany). Targeted NGS was performed using the TruSight Cancer sequencing panel (Illumina, San Diego, USA). The analyses were performed on the Illumina MiSeq® and the NextSeq system (Illumina, CA, USA). Mutation analysis was performed using NextGene and Geneticist Assistant softwares.

### Statistical Analysis

Clinicopathological characteristics and miR-34 a/b/c expression were compared by means of the non-parametric Mann-Whitney U test or Kruskal-Wallis test. Correlation analyses were assessed by Spearman-rank correlation analyses. Progression free survival (PFS) was defined as the time from diagnosis of the primary to tumor to the histopathological confirmation of recurrence or metastases and overall survival (OS) as the time from diagnosis of the primary to tumor to death from any cause or to the last clinical inspection. Univariate Kaplan-Meier analyses and multivariable Cox survival analyses were used to explore the association of miR-34 a/b/c expression or with PFS and OS. For survival analyses, patients were dichotomized into low and high mRNA-expression level groups by the optimal cut-off expression value calculated by the Youden's index based on a receiver operating characteristic curve analysis for overall survival 22. *P*-values less than 0.05 were considered as statistically significant. Statistical analysis was performed using SPSS statistical software (version 20.0.0; SPSS Inc., Chicago, IL, USA).

## Results

### Expression of miR-34a, miR-34b and miR-34c according to clinicopathological features

In 228 OC and 19 non-neoplastic fallopian tube samples, expression of miR-34 a/b/c was analysed. Significantly lower levels of miR-34 a/b/c were found in cancer specimens as compared to control tissues (*P*=0.002, *P*<0.001 and *P*<0.001, respectively; Table 1).

**Figure 1:**
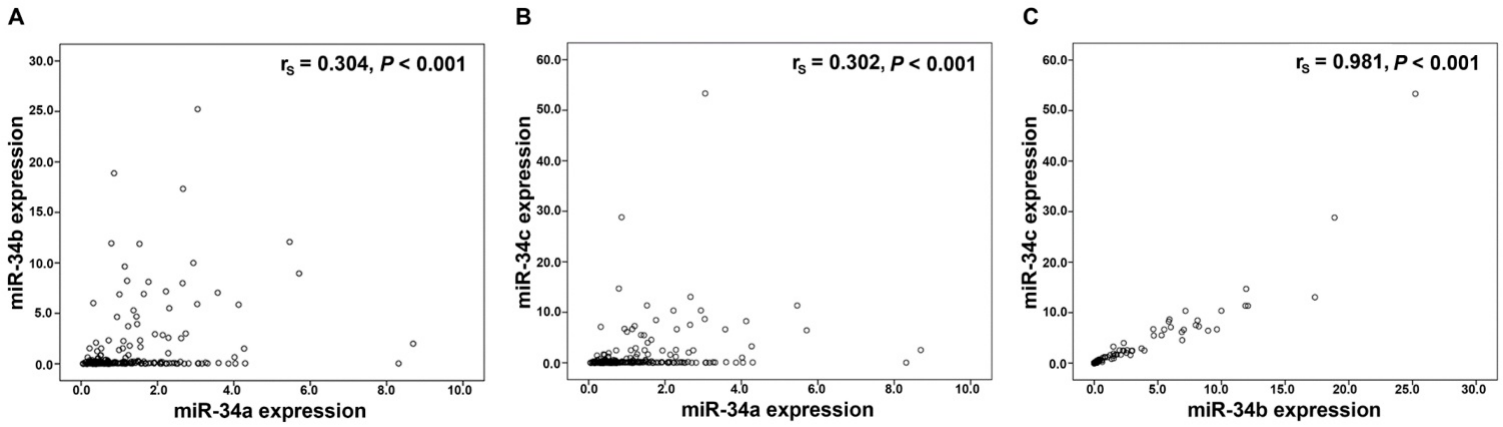
** Correlation analysis in the miR-34 family.** Linear regression analysis of **(A)** miR-34a and miR-34b expression, **(B)** miR-34a and miR-34c expression, **(C)** miR-34b and miR-34c expression. mRNA expression values were normalized to TATA box binding protein (TBP) expression.

Performing Spearman rank association analyses in the 228 OC tissues, we noted a significant and strong correlation between miR-34b and miR-34c expression (*P*<0.001; r_s_=0.981), whereas a significant but weaker correlation of miR-34a with miR-34b (*P*<0.001; r_s_=0.304) and with miR-34c (*P*<0.001; r_s_=0.302)**,** respectively was identified (Figure 1). Associations with similar correlation coefficients were also detected in control tissues (data not shown).

**Figure 2:**
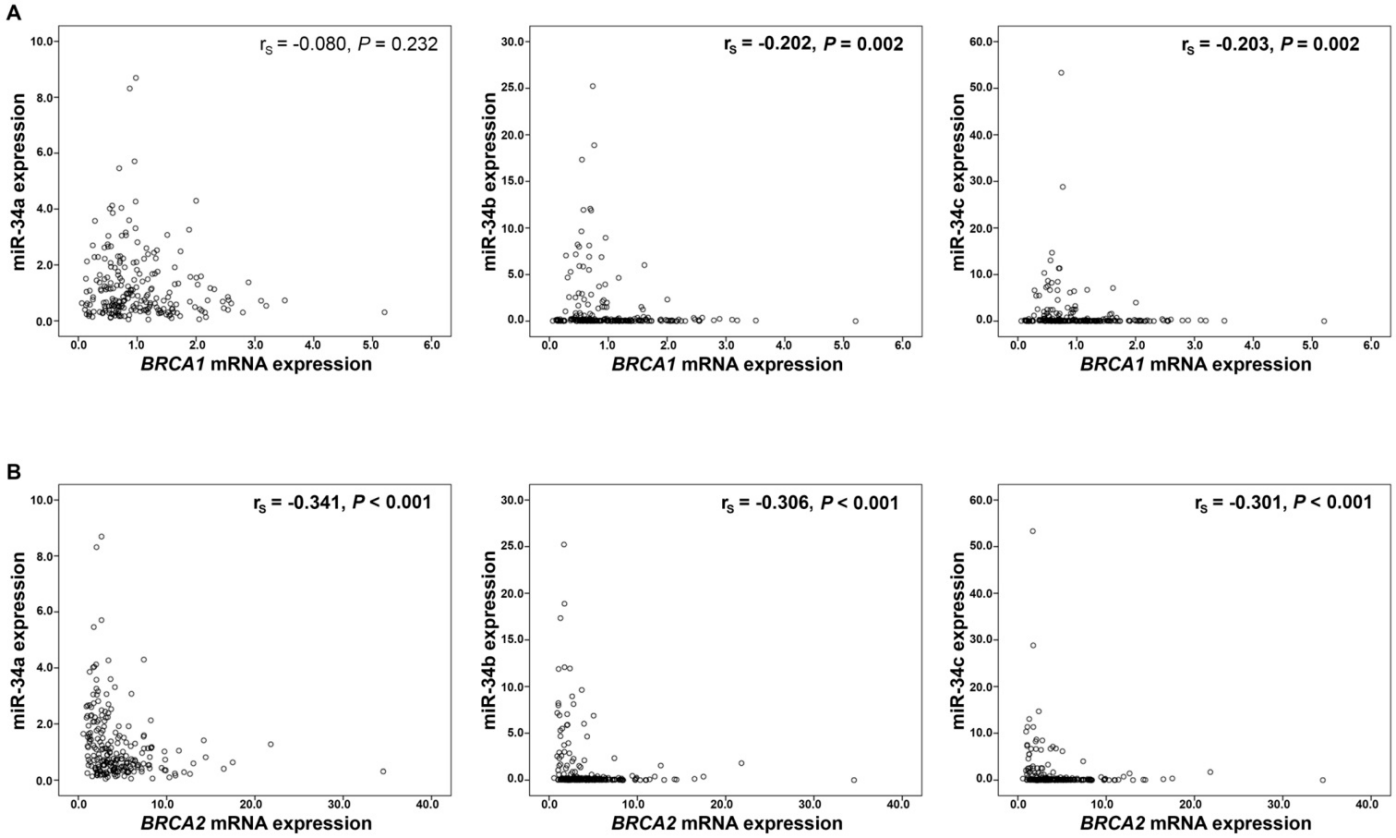
** Correlation analysis of miR-34 a/b/c and *BRCA1/2* mRNA expression.** MiR-34 expression and **(A)** *BRCA1* mRNA and **(B)** *BRCA2* mRNA expression. mRNA expression values were normalized to TBP expression.

Moreover, expression of miR-34b and miR-34c showed an inverse correlation with *BRCA1/2* mRNA expression (BRCA1: miR34b r_s_=-0.202, *P*=0.002, miR-34c r_s_=-0.203, *P*=0.002, respectively; Figure 2A; BRCA2: miR-34b r_s_=-0.306, P<0.001, miR-34c r_s_=-0.301, *P*<0.001, respectively; Figure 2B). Mir-34a however, correlated inversely only with *BRCA2* mRNA expression (r_s_=-0.341, *P*<0.001; Figure 2B). These significant associations between miR-34 members and BRCA transcripts were identified only in *BRCA1/2* wild-type cancers (data not shown).

Regarding *BRCA1/2* mutated and wild-type cancers, no significant difference in the expression of miR-34 family could be found (Table 1).

With regard to FIGO stage, expression levels of miR-34a were significantly higher in stage I/II than in stage III/IV (*P*<0.001), whereas no significant distinction between expression levels of miR-34b and miR-34c was found (Table 1).

Moreover, higher miR-34 a/b/c expression levels were observed in low grade tumors in comparison to grade 2/3 cancers (*P*=0.001, *P*=0.015 and *P*=0.012**,** respectively; Table 1).

A 1.9-fold and 1.5-fold higher miR-34a and miR-34c expression, respectively was observed in tumors which have been completely cleared during primary surgery (*P*=0.001, *P*=0.031, respectively; Table 1) in comparison to cancers resulting in any residual disease. Regarding histological subtypes, the highest expression of miR-34b and miR-34c was identified in low grade serous ovarian cancers (LGSOC) in comparison to all other histological subtypes. For miR-34a we identified a high expression in mucinous ovarian cancer, LGSOC and clear cell ovarian cancer (Table 1). Of special note was a 2.6-fold, 40.8-fold and 32.3-fold higher expression level of miR34 a/b/c**,** respectively in LGSOC compared with high grade serous ovarian cancers (HGSOC).

According to the dualistic model of carcinogenesis, we investigated the expression of the miRNAs in type 1 (n=71) and in type 2 cancers (n=157). Among all cancer samples, in type 2 ovarian cancers significantly lower expression levels of miR-34a, miR-34b and miR-34c (*P*<0.001 each) were revealed compared with type 1 cancers.

*TP53* mutated tumors exhibited significantly less expression levels of miR-34a (*P*<0.001), miR-34b (*P*=0.002) and miR-34c (*P*=0.004) in comparison with *TP53* wild-type cancers (Table 1).

In type 2 cancers no direct significant association between expression of miR-34 family members and platinum-sensitivity in the course of front-line chemotherapy was observed.

### Expression of miR-34 family members and clinical outcome

**Figure 3:**
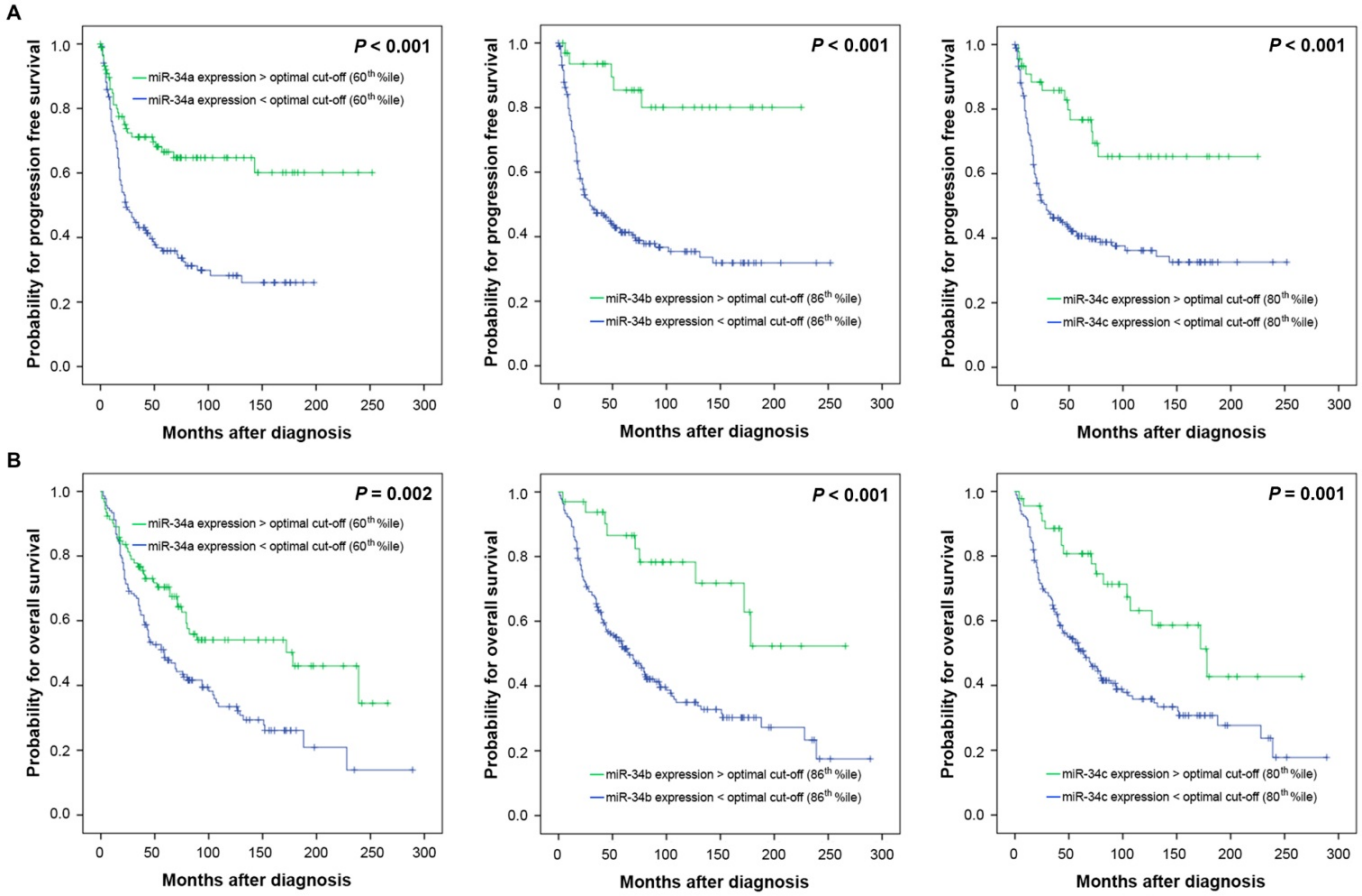
** Kaplan Meier survival analysis and miR-34 a/b/c mRNA-expression in 228 OC patients.** Progression free survival and **(A)** miR-34a expression, **(B)** miR-34b expression, **(C)** miR-34c expression. Overall survival and **(D)** miR-34a expression, **(E)** miR34b expression, **(F)** miR-34c expression. Cut-off points: miR-34a expression: low/ high: </>60^th^ percentile; miR-34b expression: low/ high: </>86^th^ percentile, miR-34c expression: low/ high: </>80^th^ percentile.

To investigate a possible biologic effect of miR-34 a/b/c on the clinical outcome, the optimal threshold for “high” and “low” expression was determined using Youden's index for progression free survival (PFS). The optimal discriminatory cut-off points corresponded to the following percentiles: miR-34a: 60^th^ percentile; miR-34b: 86^th^ percentile, miR-34c: 80^th^ percentile of the entire cohort of ovarian cancers. Univariate survival analysis revealed that high miR-34 a/b/c expression levels were associated with favourable PFS (*P*<0.001 each) and overall survival (OS) (*P*=0.002, *P*<0.001, *P*=0.001, respectively) (Table 2, Figure 3). Separate analysis of HGSOC showed a nearly identical survival outcome (PFS: miR-34a *P*=0.003, miR-34b *P*=0.001, miR-34c *P*=0.003, respectively; OS: miR-34a *P*=0.026, miR-34b *P*=0.002, miR-34c *P*=0.002, respectively) (Table 2).

Multivariate Cox regression analysis confirmed that high expression levels of miR-34 a/b/c were independently associated with favourable PFS in 228 ovarian cancer patients (miR-34a: HR 0.6 (95% confidence interval (CI) 0.4-1.0), *P*=0.033; miR-34b: HR 0.2 (95% CI 0.1-0.5), *P*=0.001 and miR-34c: HR 0.3 (95% CI 0.2-0.7), *P*=0.002, respectively) (Table 3 A) and among 128 HGSOC patients calculated separately (miR-34a: HR 0.5 (95% CI 0.2-0.9), *P*=0.019; miR-34b: HR 0.2 (95% CI 0.1-0.8), *P*=0.019 and mir-34c: HR 0.4 (95% CI 0.2-0.9), *P*=0.036, respectively) (Table 3 B). Regarding OS in the entire cohort of all ovarian cancer patients only miR-34b and miR-34c expression retained prognostic independency (miR-34b: HR 0.4 (95% CI 0.2-0.8), *P*=0.016 and mir-34c: HR 0.6 (95% CI 0.3-1.0), *P*=0.049) (Table 3 A). The separate multivariate analysis for HGSOC patients revealed also independent prognostic relevance with regard to OS for miR-34b (HR 0.3 (95% CI 0.1-0.9), *P*=0.036) and for mir-34c (HR 0.5 (95% CI 0.2-1.0), *P*=0.046) (Table 3 B).

### Validation analyses

In order to confirm our observations we analysed two publicly available datasets of OC patients recently published (GSE73581 and GSE73582) 21.

Due to limitations of availability of clinicopathological characteristics and *BRCA1/2* or *TP53* mutation data, we were not able to validate all associations identified in our study cohort.

Performing Spearman rank association analyses we confirmed the identified correlations. (GSE73581 cohort: n=168; miR-34b and miR-34c expression (P<0.001; rs=0.719), miR-34a with miR-34b (P<0.001; rs=0.827) and with miR-34c (P<0.001; rs=0.359), respectively; GSE73582 cohort: n=130; miR-34b and miR-34c expression (P<0.001; rs=0.937), miR-34a with miR-34b (P=0.008; rs=0.233) and with miR-34c (P=0.014; rs=0.216), respectively.

We used these two cohorts also to perform survival analyses to validate the prognostic relevance of the identified threshold values in our study cohort. For progression free survival independency of the prognostic value was confined to miR-34c only in the GSE73582 cohort (HR 0.5, P=0.027; Table 4). For overall survival independency was confined to miR-34a again only in the GSE73582 cohort (GSE73582: HR 0.5, P=0.046; Table 4). The separate multivariate analysis for HGSOC patients was not confirmed in both datasets (data not shown).

In a proof of concept analysis using cohort specific, optimal threshold values, we were able to confirm in both cohorts the independent prognostic relevance of miR-34b for PFS (GSE73581: HR 0.6, P=0.035; GSE73582: HR 0.6, P=0.011; Table 4). For miR-34c this was confirmed only in the GSE73582 cohort (HR 0.5, P=0.002; Table 4). The separate multivariate analysis for HGSOC patients revealed again only in the GSE73582 sample set the independent prognostic relevance for PFS of miR-34a (HR 0.4, P=0.044), miR-34b (HR 0.6, P=0.032) and miR-34c (HR 0.5, P=0.009) (data not shown).

## Discussion

In this study we could reveal significantly reduced expression of all miR-34 family members in ovarian cancer samples compared to non-neoplastic control tissues what is in accordance with previous findings 23. Moreover, the expression of miR-34 a/b/c was found to be significantly lower in *TP53* mutated samples compared with *TP53* wild type cancers. These findings confirm observations from Corney et al. 24. The lower expression in TP53 mutated tissues is probably due to the fact that all three members of the mir-34 family are under the positive regulatory control of p53 11, 14-18.

Furthermore, there is good evidence that miR-34 family is crucially involved into the ying-yang of epithelial-mesenchymal transition (EMT) and mesenchymal-epithelial transition (MET) in cancer cells. Unimpaired p53/miR-34 axis represses SNAIL which is known to induce EMT that is associated with stemness, increased migration and invasion and thus enhances the metastatic potential of malignant cells. Through this repression miR-34 family members indirectly promote MET and are abrogating malignant traits in cancer cells. In addition, in the concert of EMT regulation at least miR-34a was shown to downregulate SLUG and ZEB1 as well as several stemness factors such as BMI1, CD44, etc. On the other hand, miR-34 family members are regulated through a negative feedback loop by SNAIL and ZEB1 25-27.

Consequently, our results revealing that in FIGO stage I/II miR-34a expression levels were significantly higher than in stage III/IV of ovarian cancer seem to be in line with these findings 25, 26. Also Zhang et al. and Eitan et al. described a downregulation of miR-34 in advanced stage tumors 28, 29. Interestingly, there is data indicating that there might be a link between miR-34a and NOTCH1 expression, with respect to cancer stemness. Park et al. showed that miR-34 family was significantly reduced in chemoresistant breast cancer cells which were characterized by a higher number of cancer stem cells. In cells with reduced expression of miR-34a NOTCH1 levels have been found to be increased. After reconstitution of miR-34a, the stemness could be reduced and the sensitivity to chemotherapy was improved 30. However, this link between strong expression of miR-34a and high chemosensitivity may not be universal and the same for all tumor entities, as low grade serous ovarian cancers are constitutively chemoresistant probably due to their low proliferation rate 31-33, despite the highest miR-34 levels we measured in ovarian cancer. On the other hand, high grade ovarian cancers are generally more responsive to cytotoxic treatment 32 and were found to exhibit lower miR-34 expression in the present investigation. This was the reason we investigated in vivo sensitivity to chemotherapy in type 2 cancers only. However, in this setting none of the miR-34 family members was found to be related to the responsiveness to platinum-based primary chemotherapy.

An interesting aspect emerging from our findings was the strong correlation between miR-34b and miR-34c expression. A weaker, but nevertheless significant, correlation was identified between miR-34a and miR-34b as well as miR-34c, respectively. These findings could be explained by the fact that miR-34a is encoded by a different gene located on chromosome 1p36 while miR-34b and 34c are co-transcribed from a single gene located on chromosome 11q23 via a common promoter 11, 17. In the validation part of our study we used data from publicly available ovarian cancer patient cohorts, (GSE73581, GSE73582). Our identified correlations were confirmed in both cohorts. But the weaker correlation between miR-34a and miR-34b as well as miR-34c, respectively was observed only in the GSE73582 samples. The higher concordance between our study cohort and the GSE73582 cohort is probably due to the usage of fresh frozen tissues in both studies, whereas in the GSE7381 cohort only FFPE tissues were analysed.

P53 binding sites are present in the promoter regions of miR-34a and miR-34 b/c, capable to directly regulate the expression miR-34 family members 15, 16, 18, 34-37. Accordingly, our findings suggest significantly higher expression of miR-34a-c in type 1 cancers compared to type 2 tumors which frequently harbour *TP53* mutations 5, 7. Interestingly, miR-34a-c are known to show the most pronounced induction by TP53 out of all miRNAs 35, 38. This was proved by Corney et al., who revealed the miR-34 family to be the most suppressed miRNAs in mouse ovarian surface epithelium cells after p53 inactivation 37.

A more specified analysis according the different histological subtypes revealed the highest miR-34b and 34c levels in LGSOC in comparison to all other histological types. MiR-34a expression was however found to be high in mucinous-, low grade serous- and clear cell cancers. But in our study all clear cell cancers were high grade cancers and thus were classified as type 2 tumors and were *TP53* mutated in 31% of cases. One explanation for this contradictory finding may be the fact that especially miR-34a can be regulated by multiple p53-independent mechanisms 39.

Notably, there were significantly higher expression levels of miR-34a and 34c in tumors which have been completely cleared during primary surgery related to cancers resulting in any residual disease. These data may indicate that ovarian cancers with high miR-34a and 34c expression exhibit a lesser malignant potency with lower invasiveness and dissemination. In addition, this fact may be a major factor influencing the association between the levels of miR-34 members and the survival of OC-patients revealed in the present study. However, in multivariate Cox-regression analysis, the parameter “residual disease” was incorporated and high levels of all three members of the miR-34 family were independently associated with a beneficial PFS, as well in whole cohort of OC patients as in the subgroup of HGSOC patients. Regarding OS only miR-34b and 34c expression levels were confirmed as independent prognosticators among all OC- and HGSOC-patients. This finding is in accordance with Lee et al. who described the prognostic relevance of miR-34c in 33 HGSOC in an univariate analysis 40. Similar results for miR-34a were described in non-small-cell lung cancer by Gallardo et al. who identified miR-34a as a prognostic marker for relapse 41. The reason for the beneficial clinical outcome in patients with high miR-34 expression may be that miR-34 represses genes promoting carcinogenesis, malignant progression and stemness 11, 25, 27. As mentioned previously the transcription factor SNAIL, which is regulated by miR-34 family, is one of the key inducers of EMT 25, 26. Another crucial, cell proliferation inducing transcription factor is E2F3a, which is key activator of the cell cycle by stimulating and accelerating the G1/S transition. It has been shown that E2F3a is also negatively controlled by miR-34a. This was demonstrated in ovarian cancer cells by knock-down experiments of miR-34a which led to a significant rise of the E2F3a expression 42, 43.

In addition, L1CAM was also found to be negatively regulated by miR-34a 44. L1CAM is a functional membranous glycoprotein that confers migration and invasion properties to tumor cells and is also crucially involved in EMT 44, 45. Artificial overexpression of miR-34a in endometrial and ovarian cancer cells resulted downregulation of L1CAM expression and substantial reduction in cell migration 44. L1CAM overexpression was associated with highly impaired prognosis in several tumor entities including endometrial and ovarian cancer 46, 47. Interestingly, in ovarian cancer expression of L1CAM was linked to reduced tumor resectability at primary surgery 46. These findings 46 are corroborating the herein revealed association between residual disease and low miR-34a and miR-34c expression levels.

Regarding *BRCA1/2* mutational status, for none of the miR-34 family members a different expression between wild-type and mutated cancers was found. However, in *BRCA* wilde-type cancers, an inverse correlation between the expression of *BRCA*1*/2*-mRNA and miR-34 b/c and between miR-34a and *BRCA2* mRNA was pointed out. As the proteins encoded by the tumor suppressor genes *BRCA1/2* are profoundly involved in homologous recombination DNA repair 48, the revealed inverse associations may reflect the more pronounced malignant phenotype of cancers expressing low miR-34 family members and exhibiting a higher proliferation rates with more DNA replication. Such an increased cellular turn-over needs more DNA repair with higher expression of its main components.

The validation of the survival analyses were separated in two approaches: First, we validated our identified cutoff percentiles for miR-34 a/b/c. The independency of the prognostic value of our identified thresholds was confirmed only for PFS for miR-34c only in the GSE73582 cohort. In contrast to our data miR-34a was shown an independent factor for OS again only in the GSE73582 cohort. Second, we analysed in a proof of principle, cohort specific, optimal threshold values to confirm generally the independent prognostic relevance of the expression of each of the miR-34 family members. We were able to confirm in both cohorts the independent prognostic relevance of miR-34b for PFS. For miR-34c this was confirmed only in the GSE73582 cohort. As described above the higher concordance between our study and the GSE73582 cohort is probably due to the usage of the same sample type in both studies.

In conclusion, our findings demonstrate that miR-34 family members play an essential role in the biology of OC, especially in HGSOC. Prominent differences between the expressions of miR-34 a/b/c were pointed out for HGSOC compared with LGSOC. High miR-34 a/b/c levels were independently associated with a favourable PFS, whereas for OS prognostic independency was confined to miR-34b and 34c.

Our study clearly underscores the tumor suppressive nature of the mir-34 family members in ovarian cancer.

## Figures and Tables

**Table 1:** Association of miR-34a/b/c mRNA-expression with clinicopathological features in 228 ovarian cancer patients.

Variable	Number (percent)	mRNA expression values					
		(arbitrary units)					
		miR-34a		miR-34b		miR-34c	
		Median (IQR)	*P* value	Median (IQR)	*P* value	Median (IQR)	*P* value
**Pathology**							
ovarian cancer	228	0.75 (0.41 - 1.63)	**0.002**	0.08 (0.03 - 0.34)	**<0.001**	0.09 (0.02 - 0.45)	**<0.001**
non-neoplastic fallopian tubes	19	1.40 (1.10 - 2.39)		14.29 (9.70 - 31.71)		12.22 (3.74 - 20.46)	
**Age**							
< 61.5 years	114 (50%)	0.95 (0.39 - 1.68)	0.427	0.11 (0.03 - 1.10)	0.056	0.13 (0.03 - 1.20)	0.088
> 61.5 years	114 (50%)	0.72 (0.43 - 1.56)		0.06 (0.02 - 0.21)		0.08 (0.02 - 0.27)	
**FIGO stage**							
I/II	84 (37%)	1.27 (0.67- 2.27)	**<0.001**	0.06 (0.02 - 0.24)	0.207	0.07 (0.02 - 0.35)	0.136
III/IV	144 (63%)	0.62 (0.34 - 1.21)		0.09 (0.03 - 0.37)		0.11 (0.03 - 0.50)	
**Tumor grade**							
1	27 (12%)	1.64 (0.71 - 2.94)	**0.001**	1.51 (0.02 - 6.92)	**0.015**	1.92 (0.02 - 6.43)	**0.012**
2	117 (51%)	0.72 (0.42 - 1.61)		0.08 (0.02 - 0.39)		0.10 (0.02 - 0.54)	
3	84 (37%)	0.73 (0.32 - 1.25)		0.06 (0.03 - 0.18)		0.08 (0.03 - 0.19)	
**Residual disease**							
macroscopically tumor-free	117 (51%)	1.16 (0.49 - 2.13)	**0.001**	0.10 (0.03 - 0.72)	0.054	0.12 (0.03 - 1.10)	**0.031**
residual tumor	106 (46%)	0.62 (0.35 - 1.12)		0.06 (0.02 - 0.20)		0.08 (0.02 - 0.21)	
n.a.	5 (2%)	-		-		-	
**Histology**							
LGSOC	16 (7%)	1.64 (1.25 - 3.02)	**<0.001**	3.26 (1.02 - 7.01)	**<0.001**	2.91 (1.36 - 8.15)	**<0.001**
HGSOC	128 (56%)	0.63 (0.33 - 1.13)		0.08 (0.03 - 0.30)		0.09 (0.03 - 0.33)	
mucinous	25 (11%)	1.83 (0.72 - 2.61)		0.07 (0.02 - 1.39)		0.04 (0.02 - 1.24)	
endometrioid	45 (20%)	0.63 (0.43 - 1.46)		0.06 (0.02 - 0.22)		0.07 (0.02 - 0.27)	
clear cell	14 (6%)	1.57 (0.85- 2.57)		0.04 (0.02 - 0.11)		0.05 (0.02 - 0.11)	
**Ovarian cancer Type**							
Type 1	71 (31%)	1.53 (0.73 - 2.53)	**<0.001**	0.25 (0.04 - 4.68)	**<0.001**	0.40 (0.04 - 5.45)	**<0.001**
Type 2	157 (69%)	0.60 (0.34 - 1.15)		0.06 (0.02 - 0.18)		0.08 (0.02 - 0.19)	
***TP53* mutation**							
wild type	70 (31%)	1.68 (1.05 - 2.63)	**<0.001**	0.17 (0.03 - 2.95)	**0.002**	0.15 (0.03 - 2.62)	**0.004**
mutated	129 (57%)	0.56 (0.31 - 1.03)		0.06 (0.02 - 0.19)		0.08 (0.02 - 0.22)	
n.a.	29 (13%)	-		-		-	
***BRCA1* mutation**							
wild type	166 (73%)	0.85 (0.43 - 1.78)	0.095	0.08 (0.03 - 0.32)	0.925	0.09 (0.03 - 0.40)	0.963
mutated	33 (14%)	0.60 (0.33 - 1.20)		0.09 (0.02 - 0.25)		0.09 (0.02 - 0.44)	
n.a.	29 (13%)	-		-		-	
***BRCA2* mutation**							
wild type	178 (78%)	0.83 (0.40 - 1.65)	0.387	0.07 (0.02 - 0.32)	0.954	0.09 (0.02 - 0.43)	0.813
mutated	21 (9%)	0.72 (0.42 - 1.49)		0.09 (0.03 - 0.22)		0.15 (0.03 - 0.33)	
n.a.	29 (13%)	-		-		-	
***BRCA1/2* mutation**							
wild type	146 (64%)	0.90 (0.43 - 1.80)	0.060	0.07 (0.02 - 0.37)	0.967	0.09 (0.02 - 0.49)	0.971
mutated	53 (23%)	0.65 (0.35 - 1.22)		0.09 (0.03 - 0.23)		0.14 (0.02 - 0.33)	
n.a.	29 (13%)	-		-		-	
**Platinum sensitivity**							
refractory/ resistant	27 (17%)	0.85 (0.52 - 1.51)	0.135	0.06 (0.02 - 0.17)	0.569	0.07 (0.02 - 0.15)	0.711
very sensitive / sensitive	118 (76%)	0.58 (0.34 - 1.12)		0.06 (0.02 - 0.18)		0.08 (0.02 - 0.21)	
n.a.	10 (6%)	-		-		-	

Note: The significance level (P) was determined by Mann-Whitney U or Kruskal-Wallis test respectively. Abbreviations: HGSOC, high grade serous ovarian cancer; IQR, Interquartile range; LGSOC, low grade serous ovarian cancer

**Table 2:** ** Univariate survival analysis in 228 ovarian cancer patients.** The optimal cutoff points for miR-34a/b/c mRNA expression were calculated by the Youden's index for progression free survival (miR-34a expression: low/ high: </>60^th^ percentile; miR-34b expression: low/ high: </>86^th^ percentile, miR-34c expression: low/ high: </>80^th^ percentile).

		PROGRESSION FREE SURVIVAL			OVERALL SURVIVAL	
Variable		Median, months (95% CI)	*P* value		Median, months (95% CI)	*P* value
**Age**	< 61.5 years	57.0 (29.7 - 84.3)	0.803		129.0 (69.7 - 188.3)	**< 0.001**
	> 61.5 years	35.0 (n.r.)			49.0 (34.2 - 63.8)	
**FIGO stage**	I/II	n.r.	**< 0.001**		228.0 (61.5 - 394.5)	**< 0.001**
	III/IV	22.0 (17.0 - 27.0)			56.0 (36.2 - 75.8)	
**Tumor grade**	1	n.r.	**0.001**		239.0 (n.r.)	**0.005**
	2/3	35.0 (16.1 - 53.9)			69.0 (52.8 - 85.2)	
**Residual disease**	macroscopically tumor-free	n.r.	**< 0.001**		228.0 (79.1 - 376.9)	**< 0.001**
	residual tumor	17.0 (14.7 - 19.3)			36.0 (25.3 - 46.7)	
**Histology**	LGSOC	n.r.	**< 0.001**		n.r.	**< 0.001**
	HGSOC	24.0 (16.7 - 31.3)			52.0 (37.2 - 66.8)	
	mucinous	n.r.			172.0 (32.0 - 312.0)	
	endometrioid	n.r.			188.0 (101.5 - 274.5)	
	clear cell	18.0 (0.0-74.1)			79.0 (0.0 - 167.3)	
**Ovarian cancer Type**	Type I	n.r.	**< 0.001**		228.0 (173.8 - 282.2)	**< 0.001**
	Type II	23.0 (14.1 - 31.9)			48.0 (30.3 - 65.7)	
***TP53* aberrations**	no	n.r.	**< 0.001**		172.0 (115.1 - 228.9)	**< 0.001**
	yes	22.0 (17.0 - 27.0)			44.0 (26.6 - 61.4)	
**miR-34a mRNA expression**	low	24.0 (14.4 - 33.6)	**< 0.001**		59.0 (41.2 - 76.8)	**0.002**
	high	n.r.			178.0 (73.1 - 282.9)	
*** Subgroup analysis***						
*** HGSOC***	low	20.0 (14.4 - 25.6)	**0.003**		44.0 (35.3 - 52.7)	**0.026**
	high	143.0 (n.r.)			79.0 (54.6 - 103.4)	
*** LGSOC***	low	n.r.	0.810		*	0.379
	high	n.r.			*	
**miR-34b mRNA expression**	low	29.0 (14.0 - 44.0)	**< 0.001**		65.0 (47.0 - 83.0)	**< 0.001**
	high	n.r.			n.r.	
*** Subgroup analysis***						
*** HGSOC***	low	22.0 (17.0 - 27.0)	**0.001**		44.0 (33.8 - 54.2)	**0.002**
	high	n.r.			n.r.	
*** LGSOC***	low	24.0 (n.r.)	**0.044**		n.r.	0.972
	high	n.r.			n.r.	
**miR-34c mRNA expression**	low	29.0 (13.8 - 44.2)	**< 0.001**		64.0 (44.6 - 83.4)	**0.001**
	high	n.r.			178.0 (104.2 - 251.8)	
*** Subgroup analysis***						
*** HGSOC***	low	20.0 (15.1 - 24.9)	**0.003**		44.0 (34.0 - 54.0)	**0.002**
	high	77.0 (n.r.)			n.r.	
*** LGSOC***	low	24.0 (12.8 - 35.2)	**0.039**		*	0.427
	high	n.r.			*	

*Note*: The significance level (*P*) was determined by log-rank test. Abbreviations: n.r., not reached. *No statistics are computed because all cases are censored.

**Table 3:** ** Multivariable analysis in ovarian cancer patients.** Progression free and overall survival (A) in 228 OC patients, and (B) in 128 HGSOC patients.

**A**					
		**Progression free survival**		**Overall survival**	
**Variable**		**HR of progression (95% CI)**	***P* value**	**HR of death (95% CI)**	***P* value**
					
**Age**	low vs. high (< or > median age)	-	-	2.5 (1.7 - 3.6)	**< 0.001**
**FIGO stage**	I/II vs. III/IV	2.2 (1.2 - 4.1)	**0.009**	1.1 (0.6 - 1.8)	0.819
**Tumor grade**	1 vs. 2/3	1.6 (0.6 - 4.3)	0.330	1.5 (0.6 - 3.4)	0.365
**Residual disease**	no vs. yes	2.8 (1.7 - 4.5)	**< 0.001**	3.0 (1.9 - 4.9)	**< 0.001**
**Histology**	HGSOC vs. Others	0.8 (0.5 - 1.3)	0.374	0.8 (0.5 - 1.2)	0.210
**miR-34a expression**	low vs. high (< or > optimal cut-off)	0.6 (0.4 - 1.0)	**0.033**	0.8 (0.6 - 1.2)	0.365
**miR-34b expression**	low vs. high (< or > optimal cut-off)	0.2 (0.1 - 0.5)	**0.001**	0.4 (0.2 - 0.8)	**0.016**
**miR-34c expression**	low vs. high (< or > optimal cut-off)	0.3 (0.2 - 0.7)	**0.002**	0.6 (0.3 - 1.0)	**0.049**
					
					
**B**					
		**Progression free survival**		**Overall survival**	
**Variable**		**HR of progression (95% CI)**	***P* value**	**HR of death (95% CI)**	***P* value**
					
**Age**	low vs. high (< or > median age)	-	-	2.2 (1.4 - 3.4)	**0.001**
**FIGO stage**	I/II vs. III/IV	0.9 (0.4 - 2.0)	0.833	0.9 (0.5 - 1.7)	0.735
**Residual disease**	no vs. yes	4.0 (2.0 - 7.9)	**< 0.001**	3.3 (1.8 - 6.0)	**< 0.001**
**miR-34a expression**	low vs. high (< or > optimal cut-off)	0.5 (0.2 - 0.9)	**0.019**	0.6 (0.3 - 1.1)	0.097
**miR-34b expression**	low vs. high (< or > optimal cut-off)	0.2 (0.1 - 0.8)	**0.019**	0.3 (0.1 - 0.9)	**0.036**
**miR-34c expression**	low vs. high (< or > optimal cut-off)	0.4 (0.2 - 0.9)	**0.036**	0.5 (0.2 - 1.0)	**0.046**

Note: The significance level was determined by Cox regression analysis. HR, hazard ratio.

**Table 4:** ** Multivariable validation analysis in ovarian cancer patients.** Progression free and overall survival in 168 OC patients (GSE73581 cohort) (A), and (B) in 130 OC patients (GSE73582 cohort). The identified cutoff values from our study cohort (miR-34a: 60^th^ percentile, miR-34b: 86^th^ percentile miR-34c: 80^th^ percentile) and validation cohort specific optimal thresholds were analysed: (GSE7381: miR-34a: 49^th^ percentile, miR-34b: 24^th^ percentile, miR-34c: 7^th^ percentile; GSE7382: miR-34a: 85^th^ percentile, miR-34b: 55^th^ percentile, miR-34c: 53^rd^ percentile).

**A**					
		**Progression free survival**		**Overall survival**	
**Variable**		**HR of progression (95% CI)**	***P* value**	**HR of death (95% CI)**	***P* value**
					
**Age**	low vs. high (< or > median age)	1.5 (1.0 - 2.2)	**0.029**	1.8 (1.1 - 3.0)	**0.020**
**FIGO stage**	I/II vs. III/IV	3.1 (1.5 - 6.5)	**0.003**	2.6 (0.9 - 7.9)	0.094
**Histology**	HGSOC vs. non HGSOC	0.9 (0.6 - 1.4)	0.648	1.2 (0.7 - 2.1)	0.558
**Residual disease**	no vs. yes	**2.1 (1.4 - 3.4)**	**0.001**	**2.5 (1.3 - 4.7)**	**0.005**
**Study cohort cutoff values:**					
**miR-34a expression**	low vs. high (< or > 60^th^ percentile)	0.7 (0.5 - 1.1)	0.100	0.6 (0.4 - 1.0)	0.066
**miR-34b expression**	low vs. high (<or > 86^th^ percentile)	1.0 (0.5 - 1.7)	0.866	0.7 (0.3 - 1.6)	0.410
**miR-34c expression**	low vs. high (<or > 80^th^ percentile)	0.9 (0.6 - 1.5)	0.788	0.9 (0.5 - 1.8)	0.851
					
**Optimal cohort specific cutoff values**					
**miR-34a expression**	low vs. high (< or > optimal cut-off)	0.8 (0.5 - 1.1)	0.205	0.8 (0.5 - 1.2)	0.259
**miR-34b expression**	low vs. high (or > optimal cut-off)	0.6 (0.4 - 1.0)	**0.035**	0.6 (0.4 - 1.0)	0.062
**miR-34c expression**	low vs. high (< or > optimal cut-off)	0.7 (0.3 - 1.3)	0.238	0.8 (0.3 - 2.4)	0.742
					
					
**B**					
		**Progression free survival**		**Overall survival**	
**Variable**		**HR of progression (95% CI)**	***P* value**	**HR of death (95% CI)**	***P* value**
					
**Age**	low vs. high (< or > median age)	1.4 (0.9 - 2.1)	0.145	1.9 (1.1 - 3.5)	**0.028**
**FIGO stage**	I vs II vs III vs IV	1.4 (0.9 - 2.1)	0.090	5.8 (1.9 - 17.2)	**0.002**
**Histology**	HGSOC vs. non HGSOC	1.0 (0.6 - 1.6)	0.958	0.4 (0.2 - 1.0)	**0.038**
**Grade**	1/2 vs. 3 or undifferentiated	1.5 (0.9 - 2.5)	0.107	-	-
**Residual disease**	no vs. yes	2.6 (1.6 - 4.4)	**<0.001**	2.9 (1.4 - 5.8)	**0.004**
**Study cohort cutoff values:**					
**miR-34a expression**	low vs. high (< or > 60^th^ percentile)	0.8 (0.5 - 1.2)	0.293	0.5 (0.3 - 1.0)	**0.046**
**miR-34b expression**	low vs. high (<or > 86^th^ percentile)	0.6 (0.3 - 1.2)	0.177	0.6 (0.2 - 1.7)	0.373
**miR-34c expression**	low vs. high (<or > 80^th^ percentile)	0.5 (0.3 - 0.9)	**0.027**	0.6 (0.3 - 1.3)	0.214
					
**Optimal cohort specific cutoff values**					
**miR-34a expression**	low vs. high (< or > optimal cut-off)	0.5 (0.2 - 1.0)	0.053	0.4 (0.1 - 1.3)	0.123
**miR-34b expression**	low vs. high (or > optimal cut-off)	0.6 (0.4 - 0.9)	**0.011**	0.6 (0.3 - 1.0)	0.051
**miR-34c expression**	low vs. high (< or > optimal cut-off)	0.5 (0.3 - 0.8)	**0.002**	0.6 (0.3 - 1.1)	0.078

Note: The significance level was determined by Cox regression analysis. HR, hazard ratio.

## Abbreviations

BMI1: BMI1 proto-oncogene, polycomb ring finger
BRAF: B-Raf proto-oncogene, serine/threonine kinase
BRCA1: BRCA1, DNA repair associated
BRCA2: BRCA2, DNA repair associated
CD44: CD44 molecule (Indian blood group)
CI: confidence interval
DNA: deoxyribonucleic acid
E2F3a: E2F transcription factor 3a
EMT: epithelial-mesenchymal transition
FIGO: Fédération Internationale de Gynécologie et d'Obstétrique
HGSOC: high grade serous ovarian cancer
HR: hazard ratio
KRAS: KRAS proto-oncogene, GTPase
L1CAM: L1 cell adhesion molecule
LGSOC: low grade serous ovarian cancer
MET: mesenchymal-epithelial transition
miRNA: microRNA
NOTCH1: notch receptor 1
OC: ovarian cancer
OS: overall survival
PFS: progression free survival
SLUG: snail family transcriptional repressor 2
SNAIL: snail family transcriptional repressor 1
STIC: serous tubal intraepithelial carcinoma
TBP: TATA box binding protein
TP53: tumor protein p53
ZEB1: zinc finger E-box binding homeobox 1
